# Editorial: The bodily self in the multisensory world

**DOI:** 10.3389/fnhum.2024.1418014

**Published:** 2024-05-08

**Authors:** Carlotta Fossataro, Jean-Paul Noel, Valentina Bruno

**Affiliations:** ^1^Manibus Lab, Psychology Department, University of Turin, Turin, Italy; ^2^Center for Neural Science, New York University, New York, NY, United States

**Keywords:** bodily self representation, multisensory integration, somatosensation, sensory processing, tactile processing, proprioception, visual processing, self-other boundaries

In the dynamic relationship between our brain and the external world, the complex interaction between different sensory information shapes our perception, cognition, and even our sense of Self (Serino, 2019).

The present Research Topic investigates the relationship between sensory perception, multisensory integration, and bodily self representation. Here, the collected seven novel publications explore the fields of somatosensation, space, bodily self representation, and rehabilitation techniques, unveiling the remarkable flexibility and adaptability of the human brain in navigating the multisensory landscape. Below is a brief overview of the original research contributions that focus on the bodily self, from virtual reality experiments to clinical case studies, by offering profound insights into how our brains construct and integrate sensory experiences, ultimately reshaping our understanding of perception and consciousness.

From a clinical perspective, multisensory processing has been extensively studied. For example in neurological patients (Leo et al., 2008; Fossataro et al., 2018, 2020; Noel et al., 2019) it is established that multisensory integration fosters bodily self representation and perceptual processing, whereas alterations have been reported in psychiatric and neurodevelopmental (Noel et al., 2021; Galigani et al., 2022) conditions (Hornix et al., 2019; Gröhn et al., 2022; Brizzi et al., 2023). Here, Have et al. investigated the profound implications of brain damage on somatosensory processing. A single case sheds light on the complex relationship between vision and somatosensation, revealing a pathological imbalance in somato-representation following left parietal brain damage. Despite a preserved ability to localize tactile stimuli via motor responses, the patient exhibits deficits in discriminating stimuli applied to distal hand regions. The authors show a fascinating interaction between somatosensation and somato-representation, highlighting the pivotal role of visual information in shaping our perception of bodily sensations. In parallel, Schlienger et al. highlight the power of visuo-proprioceptive integration as a promising rehabilitation tool to alleviate phantom limb pain, and facilitate motor recovery. Exploiting the well-known mirror paradigm (Ramachandran and Rodgers-Ramachandran, 1996) in healthy participants, they demonstrate the efficacy of combining congruent visual and proprioceptive feedback to enhance movement perception of lower limbs. These findings open new avenues for therapeutic interventions, offering promising prospects for individuals coping with motor impairments. Finally, Barone et al. provide insights into the multifaceted nature of the bodily self in the context of dysregulated eating behaviors. Exploring the effect of intermittent theta burst stimulation on individuals with self-reported dysregulated eating behaviors, they showed that body dissatisfaction and drive for thinness rely on an interaction between brain laterality, cognitive processes, and emotional responses to body image. These findings could help establish asymmetric cortical excitability in the dorsolateral prefrontal cortex as a potential neurophysiological marker of dysregulated eating, and as an index in the continuum between neurotypical and neurodivergent eating behaviors.

From a developmental perspective, pivotal studies demonstrate that multisensory interactions with the external world shape the development of a coherent sense of self since birth (Orioli et al., 2018; Begum Ali et al., 2021; De Klerk et al., 2021; Ronga et al., 2021). Here, Cook et al. investigate the malleability of self-face representation through multisensory stimulation, unveiling interesting links between bodily perception and social cognition in children. By blurring the boundaries between self and other with the enfacement illusion (Tsakiris, 2008), the authors found that multisensory stimulation can significantly impact children's self-face representation and body image attitudes. Specifically, the bodily illusion leads to changes in how children perceive their own faces and bodies, as well as how they view the bodies of others, with a preference for larger body size and reduced social comparisons between self and others, suggesting an increased positive body size attitude. Interestingly, Ntoumanis et al. studied attentional engagement in human motion perception with a developmental perspective, from children to young adults. By employing naturalistic videos containing human goal-directed movement, the authors show that scenes featuring limb movements, especially simultaneous arm and leg movements, elicit higher attentional engagement than scenes without limb movement. Interestingly, the power of naturalistic limb movements in enhancing attentional engagement changes across the life span and is more pronounced in older participants, and thus can be exploited to increase the viewers' attentional focus.

Finally, by adopting different perspectives, two studies focused on how sensory information is processed and integrated in the brain. Xu et al. explore neural differences between airplane pilots and non-pilots, assuming that pilots' flight expertise leads to changes in their brain function and structure. They find that pilots' spontaneous activity in brain areas related to visual information processing and coordinated movements are significantly enhanced, suggesting that extensive training modifies bodily-related capabilities by improving monitoring, body balance, eye movement, concentration, and brain function, aiding in perception and coordination. Girondini et al. focus on how the brain processes somatosensory stimuli concerning anatomical and spatial frames of reference. Utilizing virtual reality tasks, the authors explore the impact of congruent and incongruent sensory-motor interactions on spatial perception by inducing a misalignment between the visual-motor-proprioceptive cues from body movement and the somatosensory feedback received. They spatially shift the somatosensory feedback resulting from right-hand interactions toward the opposite hand. They found that participants show a bias in left-right tactile localization, and that the perceived body-midline shifts accordingly to the location of somatosensory feedback. These findings highlight the potential for understanding spatial biases induced by sensory-motor interactions in virtual environments, contributing to advancements in bodily perception mechanisms in normal and pathological contexts.

All together, the studies of this Research Topic offer a kaleidoscopic view of bodily perception, from clinical manifestations to neuroscientific insights and social cognition processes. By embracing interdisciplinary approaches and cutting-edge methodologies, researchers continue to unravel the mysteries of the multisensory world, paving the way for transformative advancements in neuroscience and beyond.

## Author contributions

CF: Writing – original draft, Writing – review & editing. J-PN: Writing – original draft, Writing – review & editing. VB: Writing – original draft, Writing – review & editing.
